# La tuberculose nasosinusienne primaire: à propos d'un cas

**DOI:** 10.11604/pamj.2013.14.29.1039

**Published:** 2013-01-20

**Authors:** Rachid Bouchentouf, Brahim Bouaity, Mohamed Touati, Amine Benjelloun, Moulay Ali Aitbenasser

**Affiliations:** 1 Service de Pneumologie de l'Hôpital Militaire Avicenne, Marrakech, Maroc; 2 Service d'ORL de l'Hôpital Militaire Avicenne, Marrakech, Maroc

**Keywords:** Tuberculose, tuberculose extra pulmonaire, tuberculose nasosinusienne, tuberculose primaire, Tuberculosis, extra pulmonary tuberculosis, sinonasal tuberculosis, primary tuberculosis

## Abstract

La localisation nasosinusienne de la tuberculose est rare. Elle est caractérisée par une présentation clinique polymorphe et non spécifique, posant souvent un problème de diagnostic différentiel. Le diagnostic repose sur l'examen anatomopathologique et bactériologique avec examen direct et culture. Le traitement est essentiellement médical à base d'antibacillaires.

## Introduction

La tuberculose sévit toujours à l'état endémique dans les pays en voie de développement. L'agent causal est *Mycobacterium tuberculosis*. Le poumon est le lieu privilégié de la tuberculose. La localisation nasosinusienne est rare, elle est caractérisée par une présentation clinique polymorphe et non spéci'que, posant souvent un problème de diagnostic différentiel.

## Patient et observation

**Figure 1:**
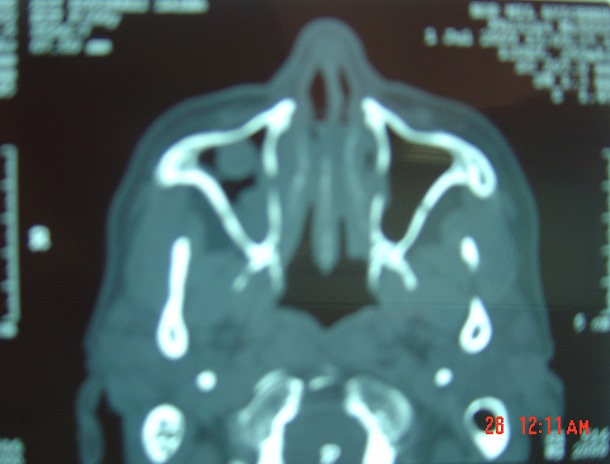
TDM du massif facial montrant un comblement du sinus maxillaire droit avec des polypes nasosinusiens

Patiente âgée de 66 ans sans antécédents pathologiques particuliers, qui présentait depuis 3 mois une rhinorrhée croûteuse avec obstruction nasale droite le tout évoluant dans un contexte apyrétique et conservation de l'état général. L'examen pleuropulmonaire était sans particularité. L'examen des aires ganglionnaires notait la présence d'adénopathie jugulocarotidienne droite de 2 cm de diamètres. Le scanner du massif facial retrouvait un comblement total du sinus maxillaire droit avec des polypes nasosinusiens (Figure 1). Le bilan biologique notait un syndrome inflammatoire avec VS à 82mm à la 1^ère^ heure, l'hémogramme note une leucopénie à 3100 éléments/mm^3^, Hémoglobine à 12g/dl, les plaquettes à 291000 éléments/mm^3^, l'IDR à la tuberculine à 15 mm.

**Figure 2:**
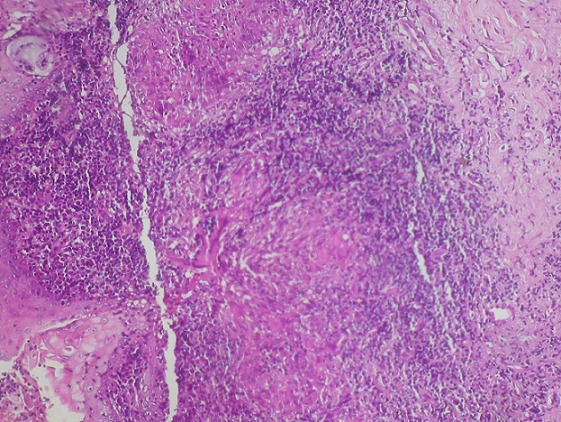
(HES x 40): muqueuse nasosinusienne siège de granulome épithélïodo-gigantocellulaire

L'endoscopie nasale montre une rhinite croûteuse avec des polypes nasosinusiens. L'examen histopathologique de la biopsie nasale met en évidence une réaction granulomateuse avec prolifération épithéloido-gigantocellulaire avec nécrose caséeuse permettant de retenir le diagnostic de tuberculose nasosinusienne (Figure 2). La radio de thorax était par ailleurs normale. Les prélèvements bactériologiques dans les crachats et les urines étaient négatifs. La patiente a béné'cié d'une quadrithérapie antituberculeuse à base d'isoniazide, rifampicine, éthambutol et pyrazinamide avec une bonne évolution clinique.

## Discussion

La tuberculose est une pandémie mondiale qui continue à défier la communauté médicale mondiale. La pauvreté et l'accroissement démographique sont les principales causes de l′éclosion et de la propagation de cette maladie. La pandémie de l′infection VIH/SIDA a contribué à l′augmentation de la diffusion de la tuberculose dans les régions du monde où le Virus de l'Immunodéficience humaine (VIH) est endémique [1]. La localisation aux voies aériennes supérieures demeure rare. Il s'agit, le plus souvent, d'une atteinte pharyngée ou laryngée. La tuberculose nasale représenterait pour Weir 2,6% des localisations extra pulmonaires [2]. La rareté de l'atteinte nasosinusienne est attribuée aux caractéristiques de la muqueuse nasale protection mécanique assurée par les mouvements ciliaires, propriétés bactéricides des secrétions nasales ainsi que la richesse lymphatique de la muqueuse pituitaire qui s'opposent au développement du Bacille de koch (BK). Mais, certains facteurs locaux (traumatismes, rhinite atrophique chronique) ou généraux (mauvaises conditions d'hygiène, immunodépression) favoriseraient le développement du BK [3]. La tuberculose nasosinusienne peut être primitive ou secondaire à une tuberculose pulmonaire. Elle se manifeste par une obstruction nasale souvent unilatérale, rhinorrhée croûteuse, épistaxis, ou masse nasale. L′examen clinique peut découvrir soit une ulcération ou un polype situé généralement dans la cloison nasale, ou au niveau du cornet inférieur [4]. Les signes radiologiques ne sont pas spécifiques. L'intérêt de l'imagerie est d'établir un bilan lésionnel et contrôle de l'évolution sous traitement.

Le diagnostic définitif de la tuberculose nasosinusienne se base sur l'histologie et sur la mise du bacille de Koch essentiellement au niveau des cultures sur le milieu Lowenstein Jensen. En cas de négativité de la culture, la PCR offre la possibilité de détecter rapidement l'antigène Mycobatérien [5]. Le diagnostic différentiel se pose avec les processus inflammatoire chronique tels la sarcoïdose, la granulomatose de Wegener, et les processus infectieux et néoplasiques [6]. Non traitée la tuberculose la tuberculose nasosinusienne se complique de perforation septale, de rhinite atrophique ou de sténose nasale. Le traitement est essentiellement médical à base d'antibacillaires permettant souvent la guérison complète.

## Conclusion

La tuberculose nasosinusienne est souvent de diagnostic difficile. Il faudrait l'évoquer devant tout signe rhinologique inexpliqué et résistant au traitement habituel. Son pronostic est favorable sous une antibiothérapie antituberculeuse classique et précoce.

## References

[1] Slutsker L, Castro KG, Ward JW, Dooley SW (1993). Epidemiology of extra pulmonary tuberculosis among persons with AIDS in the United States. Clin Infect Dis..

[2] Contant A, Fantan Y, Peraldi R, Acquaviva F (1995). Tuberculose nasale: A propos d'un cas. Rev Laryngol Otol Rhinol..

[3] Hughes RG, Drake-Lee A (2001). Nasal manifestations of granulomatous disease. Hosp Med..

[4] Butt AA (1997). Nasal tuberculosis in the 20th century. Am J Med Sci..

[5] Clarridge JE, Shawar RM, Shinnick TM (1993). Large-scale use of polymerase chain reaction for detection of Mycobacterium tuberculosis in a routine mycobacteriology laboratory. J Clin Microbiol..

[6] Baruah B, Goyal A, Shunyu NB, Lynrah ZA, Raphael V (2011). Tuberculosis of nose and palate with vanishing uvula. Indian J Med Microbiol..

